# Giant Trichobezoar of Duodenojejunal Flexure: A Rare Entity

**DOI:** 10.4103/1319-3767.70627

**Published:** 2010-10

**Authors:** Vipul D. Yagnik, Bhargav D. Yagnik

**Affiliations:** Ronak Endo-laparoscopy and General Surgical Hospital, Patan, India. E-mail: vipul.yagnik@gmail.com; 1 General practitioner, Ahmedabad, Gujarat, India

Sir,

We read the article entitled “Giant trichobezoar of duodenojejunal flexure: A rare entity”[1] with interest. Isolated involvement of duodenojejunal flexure is an unusual finding and are few cases reported in the literature. The correct Persian word for Bezoar is ‘padzehr’[2] not ‘panzehar’.[1] We would like to add few interesting and important information regarding Bezoar and Rapunzel syndrome which may be of help to the readers. Rapunzel was a long haired girl in Grimm’s fairy tales who was imprisoned in a tall castle and lowered her hair from a high window to allow her prince to climb up and rescue her.[2] While reporting a case, it is better to mention history in detail. In this particular case, authors should have at least mentioned whether history of trichophagia was present or not. One interesting fact about Rapunzel syndrome is that only two cases have been reported in the male. Male patients were reported by Hirugade *et al*.[3] and Dinyal *et al*.[4] The male patient reported by Hirugade *et al*.,[3] had a habit of eating the hair of his sister and Dinyal *et al* with ‘comma sign’. Few more predisposing conditions in addition to those mentioned by the authors are diabetes mellitus, mixed connective tissue disease, and hypothyroidism. Various new techniques, such as water jet, Dormia basket, polypectomy snare, Nd:YAG laser, Bezotome and modified lithotripter, have been developed to treat the bezoars with variable success rates. After removal, prevention of recurrence is important. Prophylactic oral enzyme andprokinetic along with psychotherapy and regular follow up may help in preventing recurrence.[2]
